# The dynamics of state math anxiety vary by paradigm and timing during arithmetic

**DOI:** 10.1038/s41539-025-00398-z

**Published:** 2026-01-20

**Authors:** Xinru Yao, Julia F. Huber, Zixu Li, Yaren Findik, Hans-Christoph Nuerk, Christina Artemenko

**Affiliations:** 1 Department of Psychology, University of Tuebingen, Tuebingen, Germany; 2 LEAD Graduate School and Research Network, University of Tuebingen, Tuebingen, Germany; 3 Department of Social Work and Social Administration, University of Hong Kong, Hong Kong, China; 4 German Centre for Mental Health (DZPG), Tuebingen, Germany

**Keywords:** Mathematics and computing, Neuroscience, Psychology, Psychology

## Abstract

Math anxiety impairs performance, but how its state and trait components interact with task characteristics remains unclear. We examined how state math anxiety varies as a function of trait math anxiety, task paradigm, and temporal dynamics, and how trait math anxiety relates to arithmetic performance. Results revealed that production paradigms, which require generating answers, elicited higher state math anxiety compared to decision paradigms, particularly for individuals with high trait math anxiety. Looking into different task phases, state math anxiety decreased during arithmetic due to habituation and after arithmetic due to relief. Additionally, the anxiety-complexity effect was replicated: Individuals with higher trait math anxiety were slower in solving complex arithmetic with a carry or borrow operation. This study confirmed the situation-dependent characteristics of state math anxiety and its dependency on paradigm and trait math anxiety, with implications for designing interventions that mitigate anxiety and optimize learning.

## Introduction

Mathematics is a fundamental subject in education and an essential skill for daily life. From science and engineering to finance and technology, mathematical skills are needed for numerous fields, making proficiency in math crucial for academic and professional success. Studies have consistently shown that math skills are linked to problem-solving skills, logical thinking^1,2^, and career opportunities, especially in STEM fields^3^. However, despite its importance, mathematics often provokes negative emotions, particularly math anxiety, which can significantly influence performance and long-term academic outcomes due to avoidance of the subject.

Math anxiety, characterized by tension and fear that interfere with manipulating numbers and solving mathematical problems^4^, has long been a topic of significant interest in both psychological and educational research. Math anxiety has consistently been linked to poor math performance^5,6^. Moreover, an anxiety-complexity effect was found, indicating that math-anxious individuals have difficulties particularly with more complex arithmetic^4,7,8^. This means that task difficulty affects performance in individuals with higher levels of anxiety more than in individuals with lower levels of anxiety. Several theoretical accounts have been proposed to explain the relationship between math anxiety and performance, which can be broadly categorized into cognitive disruption explanations, competency-based explanations, and integrative approaches.

The Disruption Account^9,10^ postulates that math anxiety triggers intrusive thoughts and ruminations, which occupy working memory resources, reduce cognitive efficiency and subsequently impair math performance. This explains the anxiety-complexity effect, as more complex arithmetic (such as addition with carry operation and subtraction with borrow operation) relies on working memory^11^ and thus is even more impaired than performance in simple arithmetic. However, recent findings from network analysis suggest that math anxiety and working memory are independently linked to math performance, indicating that the relationship between math anxiety and performance is not solely dependent on working memory^12^. Further support for the Disruption Account is provided by the Processing Efficiency Theory and Attentional Control Theory. The Processing Efficiency Theory^13^ suggests that anxiety reduces the efficiency (not effectiveness) of cognitive processing by transferring attentional resources to task-irrelevant worry, thereby leaving fewer resources available for task performance. The Attentional Control Theory^14^ further emphasizes that anxiety disrupts the balance between goal-directed (top-down) and stimulus-driven (bottom-up) attentional control, leading to impaired concentration and thus impairing task performance.

While the Disruption Account emphasizes how anxiety impairs performance through cognitive mechanisms, the Reduced Competency Account^10^ offers an alternative perspective, suggesting that math anxiety is the result of poor math ability, where reduced competency leads to difficulties in learning and performance, ultimately causing anxiety, with individuals often avoiding math-related tasks and opportunities for improvement^15–17^. An extreme example of this is that children with dyscalculia exhibit higher levels of math anxiety compared to typically developing children^18^. Extending the Reduced Competency Account, the Interpretation account^10^ argues that math anxiety stems not only from poor math skills or negative experiences but from how individuals interpret and appraise their math-related experiences.

Rather than viewing these accounts as mutually exclusive, all different theoretical accounts can be integrated in a reciprocal theory, stating a bidirectional relationship between math anxiety and math performance. Accordingly, math anxiety and mathematics performance influence each other resulting in a vicious cycle^19,20^. Taken together, math anxiety shapes and is shaped by performance and experiences in math.

Based on the state-trait anxiety model^21^, two distinct forms of math anxiety can be differentiated^22^: On the one hand, trait math anxiety refers to a stable, enduring personality trait reflecting a general tendency to feel anxious during math-related tasks. On the other hand, state math anxiety refers to a temporary, situation-specific form of anxiety that arises in response to math tasks. State math anxiety can be accompanied by physiological responses such as heightened autonomic nervous system arousal^20,22^. Crucially, state math anxiety represents the dynamic, moment-to-moment emotional experience during actual mathematical engagement, making it particularly relevant for understanding real-time cognitive processing and performance.

The relationship between trait and state math anxiety is complex. Individuals with high trait math anxiety are more likely to experience high state anxiety during math tasks^23^, though the two forms of anxiety are shaped by different factors. Trait math anxiety is influenced by past experiences and long-term beliefs about math, while state math anxiety is sensitive to immediate contexts and specific task characteristics^24–27^. Task difficulty can be considered as a situational determinant of state math anxiety, with more difficult math problems being associated with higher levels of state anxiety in children^27,28^ and adults^24,29,30^. The interplay between state and trait math anxiety and their combined impact on real-time math performance is a central question. Some studies propose that state math anxiety directly mediates the negative effect of trait math anxiety on performance, particularly under demanding conditions^31^. However, Pelegrina et al.^32^ found that when both types of math anxiety were examined simultaneously, trait math anxiety was a stronger predictor of math performance than state math anxiety, with the effects of state math anxiety largely attributable to shared variance with trait math anxiety.

Therefore, to accurately assess math anxiety during arithmetic tasks, it is essential to consider both trait and state math anxiety. Trait assessments of math anxiety are designed to measure traits, i.e., stable predispositions of an individual shown across many situations, but not situational fluctuations. As trait assessments are often based on hypothetical or retrospective questionnaires, there might be a tendency to overestimate the anxiety experienced in mathematical situations due to the intensity bias^33–35^ and a bias due to beliefs about one’s own competence in math^34^. In contrast, state questionnaires rather directly assess emotions in a certain situation and thus provide a more accurate reflection by capturing emotions in real-time. Furthermore, state math anxiety can be captured through different modalities, such as self-report and physiological measures, which may provide distinct information and relate differently to trait measures^30,31^. This leads to discrepancies between trait and state math anxiety^34,36^ and highlights the value of state assessments for math anxiety^24^.

In distinguishing between trait and state math anxiety, other individual differences – such as test anxiety, math self-concept, math ability, working memory capacity, and gender – may also play a role^24,37^. However, the present study focuses primarily on state math anxiety and its dependance on trait math anxiety.

As state math anxiety is sensitive to situational fluctuations, the way math performance is assessed might impact state math anxiety. Math performance can be assessed in several ways: Children in school almost exclusively solve mental arithmetic in production paradigms with an open answer format, whereas studies in laboratory settings often use decision paradigms with given answers. Studies have shown that children’s performance can vary depending on the response format, with better performance observed in decision paradigms compared to production paradigms^38,39^. Similarly, an experiment in adults showed that decision paradigms (e.g., verification, forced-choice, and delayed forced-choice) lead to better performance compared to production paradigms (e.g., written production, verbal-keyboard production, and simple verbal production)^40^. Thus, the response format of an arithmetic task creates different situations so that performance varies with paradigm.

Moreover, math performance is related to math anxiety. This raises the question of whether (state) math anxiety also varies across different paradigms. Given that state math anxiety is sensitive to the specific situational context, it is plausible that varying paradigms, each involving distinct solution processes and different levels of uncertainty about mistakes and failures, may impact state math anxiety and its relationship to performance. The open format of response and the generally lower performance observed in production paradigms may lead to more anxiety compared to decision paradigms where possible responses are given, as production paradigms place higher calculation demands and increase uncertainty about correctness. Therefore, the first objective of this study is to evaluate whether state math anxiety depends on the paradigm.

Additionally, the relationship between speed and accuracy can vary with math anxiety levels: compared to individuals with low math anxiety, who are fast and accurate in arithmetic, individuals with moderate math anxiety are slower and individuals with high math anxiety are less accurate^4^. Consequently, there is a need to investigate whether specific speed-accuracy trade-offs can be observed due to math anxiety. The speed-accuracy trade-off is a strategic adjustment in the decision process that adapts to environmental demands^41,42^. As math-anxious individuals may prioritize speed or accuracy differently depending on the paradigm, potentially influencing their overall performance, the potential for speed-accuracy trade-offs needs to be explored.

When investigating state math anxiety, it is important to account for possible fluctuations throughout a mathematical task. Research shows that state anxiety anticipated before a math task can negatively affect performance. For example, Orbach and Fritz^43^ found that children’s math performance was impacted by state anxiety before – but not after – completing math tasks^44,45^. Similarly, Goetz et al.^46^ observed higher levels of state anxiety before a test compared to afterwards, suggesting that the anticipation of the task plays a significant role. Taken together, these results suggest that state math anxiety is higher when anticipating a math task compared to the relief after completing the math task.

Conversely, Conlon et al.^47^ found that state math anxiety could increase after the math task, particularly after challenging problem-solving tasks, reflecting the cognitive demands and complexity of the task. Supporting this evidence, physiological markers such as heart rate and skin conductance revealed that state math anxiety increased during a math exam, especially in later stages, likely due to rising time pressure and task difficulty^48^. These seemingly contradictory findings highlight the complexity of state math anxiety and its temporal dynamics, raising the question: How does the level of state math anxiety change during math tasks? Is it elevated or reduced compared to the pre-task level?

Resolving these inconsistencies requires a clearer understanding of how specific phases of a task (i.e., pre-task anticipation, mid-task progression, and post-task evaluation) contribute to state math anxiety. Research on anxiety therapies, such as exposure techniques, shows that state anxiety usually decreases over time due to the process of habituation^49^. However, this general decrease over time overlaps with the mid-task and post-task phases, making it unclear what exactly causes the reduction in anxiety: Is it only the relief after task completion that reduces anxiety, or rather the repetitive exposure to the task? This is another objective of the current study. To distinguish whether these temporal patterns are specific to math anxiety or reflect broader anxiety dynamics during task performance, the present study also assessed general state anxiety.

The present study aims to provide a comprehensive understanding of the dynamics of math anxiety and its impact on arithmetic performance, considering both paradigm effects and temporal trends. Specifically, we examined how state math anxiety varies as a function of trait math anxiety, depending on task paradigm and on time, respectively. Additionally, we further evaluated the relation of trait math anxiety to performance in this context. The following conceptual hypotheses were preregistered (https://aspredicted.org/gf8dc.pdf) before data collection:

For math anxiety differences between paradigms (H1), (H1a) state math anxiety across paradigms (confirmatory)—we expect state math anxiety to be higher in production paradigms, where participants must generate the answer themselves, compared to decision paradigms, where the correct answer is selected from given options. Additionally, higher trait math anxiety is expected to be associated with a larger difference in state math anxiety between the paradigms (interaction between paradigm and trait math anxiety). (H1b) trait math anxiety and arithmetic performance (confirmatory & exploratory)—we expect a negative relation between trait math anxiety and arithmetic performance, with higher math anxiety associated with longer response times and lower accuracy. Additionally, we will explore whether the relation between trait math anxiety and arithmetic performance differs between production and decision paradigms.

For temporal dynamics of state math anxiety (H2), (H2a) changes across phases of the task (exploratory)—we will explore changes in state math anxiety across different phases of an arithmetic task (pre-, mid-, post-task), i.e., how state math anxiety changes from before to during and after arithmetic. Additionally, we will examine how trait math anxiety influences these changes in state math anxiety across the task phases. (H2b) detailed dynamics during the task (exploratory)—we will further explore the temporal dynamics of state math anxiety in more detail during the arithmetic task, using six measurement time points taken during breaks at the midpoint of each paradigm block. This detailed analysis is based on an additional related preregistration (https://aspredicted.org/sc87-3ntj.pdf), which was completed after data collection but before any data inspection or analysis regarding this hypothesis.

Regarding the relation between trait math anxiety and performance (H3), (H3a) anxiety-complexity effect (confirmatory)—an anxiety-complexity effect is expected regardless of the paradigm, i.e., higher levels of trait math anxiety are associated with larger carry/borrow effects. (H3b)speed-accuracy trade-off (exploratory)—for trait math anxiety, we will further explore potential speed-accuracy trade-offs within and between subjects in the different paradigms.

The operationalization of the study considers measures of trait and state math anxiety while participants are performing an arithmetic task. Trait math anxiety (AMAS) was assessed only before the arithmetic task, while state math anxiety (SMA) and state anxiety (STAI-SKD) were assessed before, during and after the arithmetic task. The arithmetic task consisted of two-digit addition and subtraction problems presented in different paradigms (decision vs. production). Arithmetic performance outcomes were accuracy (ACC) and response time (RT).

## Results

For data analysis, paradigm effects (H1) and temporal dynamics (H2) were examined in separate models as they address conceptually distinct research questions. Results for the anxiety-complexity effect (H3a) and speed-accuracy trade-off (H3b) can be found in Table S2. Participants’ mean trait math anxiety was 1.98 (*SD* = 0.68). Descriptive analysis results were shown in Table 1 and Table S1.

**Table 1:** Descriptive statistics of state (math) anxiety measures across task phases

Category	Temporal progression	State math anxiety	State anxiety
three task phases	pre-task	2.06 (1.05)	1.82 (0.75)
	mid-task	1.66 (0.69)	1.61 (0.57)
	post-task	1.46 (0.69)	1.29 (0.51)
mid-task measurement time	1	2.01 (0.76)	1.84 (0.79)
	2	1.84 (0.86)	1.71 (0.74)
	3	1.86 (0.92)	1.76 (0.82)
	4	1.63 (0.79)	1.54 (0.74)
	5	1.49 (0.68)	1.42 (0.62)
	6	1.50 (0.69)	1.42 (0.61)
paradigms	decision	1.56 (0.61)	1.45 (0.54)
	production	1.88 (0.76)	1.78 (0.64)

*Notes.* Values are presented as *M* (*SD*) for each measure. State math anxiety was measured with the 1-item version (SMA-1) before and after the arithmetic task.

### Paradigm-dependent analysis for state math anxiety

Regarding H1a, an LMM with state math anxiety as the dependent variable was conducted including fixed effects for paradigm (production vs. decision), trait math anxiety, and their interaction. The LMM further included a random intercept for subject (but not – as incorrectly preregistered – for item, because state measures were not assessed at an item level but only at a block level). Additionally, the LMM did not include a random slope for paradigm, as specified in the preregistration, because incorporating this would have made the random effects structure too complex given the limited number of observations.

**Fig. 1:**
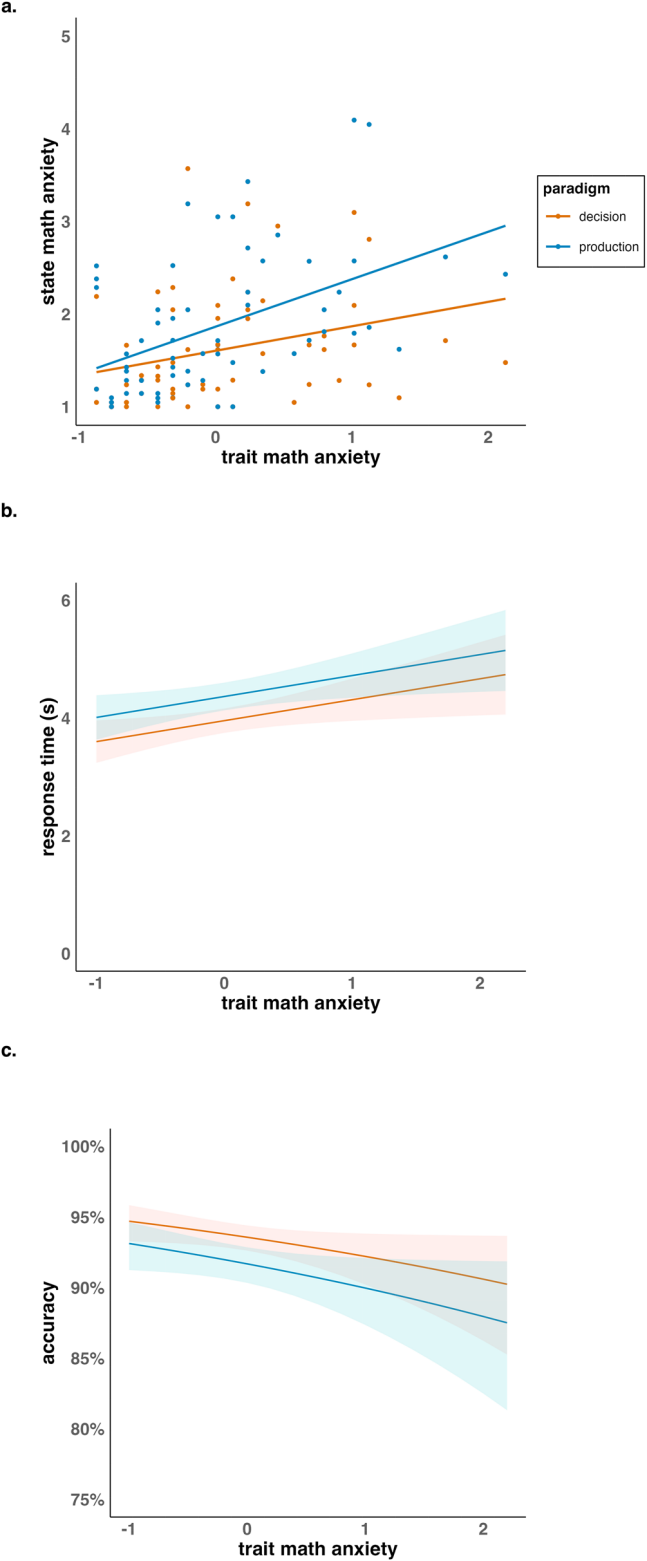
Relation of trait math anxiety to state math anxiety and performance dependent on paradigm. **a** shows the interaction between paradigm and trait math anxiety on state math anxiety. **b**, **c** show the effects of paradigm and trait math anxiety on performance in terms of response time and accuracy, respectively. Response time analyses were based on correctly solved trials only.

The final LMM (Model A) included fixed effects for paradigm, trait math anxiety and their interaction, with a random intercept for subject (see Table 2, Fig. 1a). The main effect of paradigm indicates that state math anxiety is higher in production paradigms compared to decision paradigms, with an estimated increase of 0.29. The main effect of trait math anxiety indicates that state math anxiety increases with increasing trait math anxiety, with an estimate of 0.28. The interaction of paradigm and trait math anxiety indicates that the effect of trait math anxiety on state math anxiety is larger in production than in decision paradigms, by an estimate of 0.24. These results imply that state math anxiety is higher in production than in decision paradigms, particularly for individuals with higher trait math anxiety.

**Table 2:** LMM/GLMM results

Predictors	*β*	*CI*	*t / z*	*p*	*R²*
**Model A**: LMM for paradigm and trait math anxiety on state math anxiety					0.87
(intercept)	1.57	1.42 – 1.72	20.74	**< 0.001**	
paradigm	0.29	0.21 – 0.38	6.64	**< 0.001**	
trait math anxiety	0.28	0.06 – 0.50	2.52	**0.013**	
paradigm × trait math anxiety	0.24	0.11 – 0.37	3.64	**< 0.001**	
**Model B**: LMM for trait math anxiety and paradigm on response time					0.42
(intercept)	3.96	3.75 – 4.16	37.58	**< 0.001**	
paradigm	0.41	0.30 – 0.52	7.43	**< 0.001**	
trait math anxiety	0.36	0.06 – 0.65	2.38	**0.017**	
*paradigm × trait math anxiety*					
**Model C**: GLMM for trait math anxiety and paradigm on accuracy					0.15
(intercept)	2.68	2.53 – 2.82	35.42	**< 0.001**	
paradigm	-0.28	-0.40 – -0.16	-4.46	**< 0.001**	
trait math anxiety	-0.21	-0.41 – 0.00	-1.93	0.053	
*paradigm × trait math anxiety*					
**Model D**: LMM for three-time points analysis on state math anxiety					0.70
(intercept)	1.73	1.59 – 1.87	23.95	**< 0.001**	
time	-0.30	-0.39 – -0.22	-7.32	**< 0.001**	
trait math anxiety	0.60	0.39 – 0.81	5.63	**< 0.001**	
time × trait math anxiety	-0.31	-0.43 – -0.19	-5.07	**< 0.001**	
*time*^2^					
*time*^2^ *× trait math anxiety*					
**Model E**: LMM results for six-time points analysis on state math anxiety					0.75
(intercept)	1.72	1.48 – 1.96	14.03	**< 0.001**	
time	-0.11	-0.13 – -0.08	-8.96	**< 0.001**	
trait math anxiety	0.40	0.18 – 0.62	3.65	**< 0.001**	
time × trait math anxiety	-0.07	-0.11 – -0.04	-3.96	**< 0.001**	
*time*^2^					
*time*^2^ *× trait math anxiety*					

*Notes*. All models shown here are reduced models. *t* value for LMM and *z* value for GLMM. Bold values indicate statistical significance. Time and trait math anxiety were centered. Paradigm was dummy coded with decision paradigm as reference for production paradigm. Conditional *R²* quantifies the proportion of variance explained by the entire model, including both fixed and random effects. Excluded factors from the full models are shown in italics. Full model specifications and model selection procedures are detailed in Table S3.

### Paradigm-dependent analysis for arithmetic performance

Regarding H1b, an LMM with RT as the dependent variable and a GLMM with ACC as the dependent variable were conducted including fixed effects for paradigm, trait math anxiety, and their interaction. The (G)LMM further included random intercepts for both subject and item as well as a random slope for paradigm.

The final LMM for RT (Model B) included fixed effects for trait math anxiety and paradigm, with random intercepts for subject and item as well as a random slope for paradigm (see Table 2, Fig. 1b). The main effect of paradigm indicates that arithmetic in production paradigms needs longer by an estimate of 0.41 s to be solved than in decision paradigms. The main effect of trait math anxiety indicates that for every unit increase in trait math anxiety, the response time increases by an estimate of 0.36 s, so that individuals with higher trait math anxiety take longer to solve arithmetic.

Similar to RT, the final GLMM for ACC (Model C) included fixed effects for trait math anxiety and paradigm, with random intercepts for subject and item as well as a random slope for paradigm (see Table 2, Fig. 1c). The main effect of paradigm indicates that accuracy was lower by an estimate of -0.28 in production compared to decision paradigms. The main effect of trait math anxiety was marginally significant with an estimate of -0.21, indicating that individuals with higher math anxiety tend to make more errors in arithmetic.

Together, the results suggest that individuals with higher trait math anxiety show worse arithmetic performance, and production paradigms are more difficult than decision paradigms.

### Time analysis pre-, mid-, and post-arithmetic task

Regarding H2a, LMMs with state math anxiety (SMA-1) and state anxiety as dependent variables were conducted including fixed effects for both the linear (time) and quadratic term of time (time²), trait math anxiety, the interaction between time and trait math anxiety, and the interaction between time² and trait math anxiety. The linear term of time includes 3 time points: pre-, mid- (average across 6 measurement points) and post-arithmetic task. The quadratic term of time was introduced to account for non-linear trajectories, for example, an initial increase in anxiety during the task followed by a decrease at the end after the task is successfully completed. The LMMs further included a random intercept for subject.

**Fig. 2:**
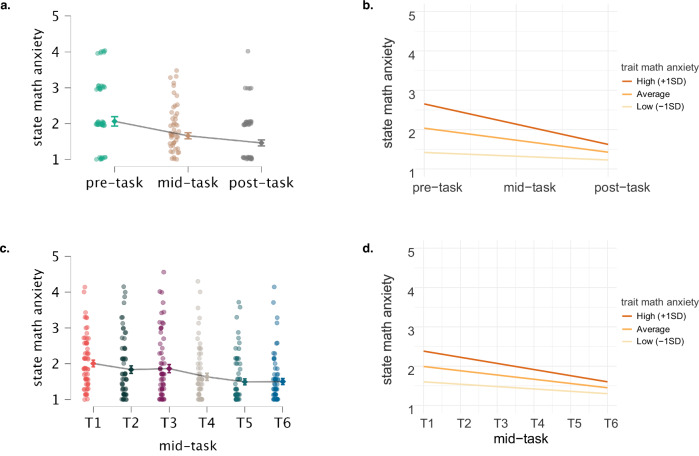
State math anxiety changes across different task phases and measurement times. **a**, **c** show the decrease in state math anxiety across the three task phases and across the six measurement times during the arithmetic task, respectively. Error bars represent the standard error of the mean (*SEM*). **b**, **d** show a simple slope analysis for state math anxiety depending on trait math anxiety (average level, high level with 1 *SD* above the average, and low level with 1 *SD* below the average) across the three task phases and across the six measurement times during the arithmetic task. State math anxiety was measured via questionnaires and therefore reflects all participants regardless of their individual performance.

The final LMM model on state math anxiety (Model D) included fixed effects for time, trait math anxiety, and the interaction between time and trait math anxiety, with a random intercept for subject (see Table 2, Fig. 2a & b). The main effect of time indicates that state math anxiety decreases over time by an estimate of -0.30 per time point. The main effect of trait math anxiety indicates that for each unit increase in trait math anxiety, state math anxiety increases by an estimate of 0.60, so that individuals with higher trait math anxiety show higher levels of state math anxiety. The interaction effect of time and trait math anxiety with an estimate of -0.31 indicates that the decrease in state math anxiety over time is stronger for individuals with higher trait math anxiety. A similar analysis was conducted with state anxiety as a dependent variable (for results see Fig. S1).

### Time analysis during the arithmetic task

Regarding H2b, LMMs with state math anxiety (SMA) and state anxiety as dependent variables were conducted including fixed effects for time (6 measurement times during arithmetic), the quadratic term of time (time²), trait math anxiety, the interaction between time and trait math anxiety, and the interaction between time² and trait math anxiety. The LMMs further included random intercepts for subject and paradigm. Paradigm was included as a random effect to account for paradigm-specific effects.

The final LMM model (Model E) included fixed effects for time, trait math anxiety, the interaction between the time and trait math anxiety, with random intercepts for subject and paradigm (see Table 2, Fig. 2c & d). The main effect of time indicates that for each repetition of the arithmetic task, state math anxiety decreases over time by an estimate of -0.11. The main effect of trait math anxiety indicates that for each unit increase in trait math anxiety, state math anxiety increases by an estimate of 0.40, so that individuals with higher trait math anxiety show higher levels of state math anxiety. The interaction of time and trait math anxiety with an estimate of -0.07 indicates that, as time progresses, individuals with higher trait math anxiety tend to experience a decrease in state math anxiety at a slightly faster rate compared to those with lower trait math anxiety. This suggests that while initial anxiety levels may be elevated, individuals with higher trait math anxiety may adapt or regulate their state math anxiety over the progress of the task. A similar analysis was conducted with state anxiety as dependent variable (for results see Fig. S1).

## Discussion

This study explored the dynamics of state math anxiety across different paradigms and its relation to trait math anxiety and arithmetic performance. The findings highlight three key insights: (1) Production paradigms elicit higher state math anxiety than decision paradigms, especially for individuals with higher levels of trait math anxiety. (2) Higher trait math anxiety is associated with worse performance, especially in production paradigms. (3) State math anxiety decreases over time from before to after the arithmetic task as well as during the arithmetic task, especially for individuals with higher trait math anxiety who start with higher levels of state math anxiety, suggesting habituation and regulation mechanisms in managing anxiety.

The response format in math tasks indeed influences emotional states. In line with the interaction model of anxiety^50^, situational characteristics such as paradigm can modulate momentary (state) anxiety. Higher state math anxiety in production paradigms compared to decision paradigms suggests that generating answers, rather than choosing from answers, imposes greater emotional demands. In decision paradigms, performance is better and various strategies can be used to solve arithmetic^40^. For instance, estimation strategies, verifying the correctness of a given answer, or rejecting incorrect answers contribute to more efficient and accurate task performance^51,52^. Better task performance was also shown to be associated with lower state math anxiety^24^, so that the paradigm effect on state math anxiety might be driven by performance differences. In contrast, production paradigms require individuals to generate answers without the aid of pre-existing choices, like in real world contexts. This demands a higher cognitive load and is associated with less security, leading to higher state math anxiety, as found in the current study. In sum, production paradigms in arithmetic increase difficulty and state math anxiety compared to decision paradigms.

Notably, the production-induced increase in state math anxiety was predicted by trait math anxiety. Individuals with higher trait math anxiety are particularly sensitive to the cognitive demands imposed by the production paradigm, and thus experience especially more state math anxiety when they are required to exactly calculate the result without checking preexisting solutions. Previous research^7,53^ has primarily demonstrated that high trait math anxiety reduces performance, particularly under complex task conditions, reflecting the anxiety-complexity effect, which is replicated in the current study^4,7,8^. Our findings show that state math anxiety is similarly affected by the interplay of trait math anxiety and complex task demands (by paradigm). However, state math anxiety might be rather an accompanying phenomenon for affective experiences in a situation (as measured by state math anxiety) than a factor that further impairs performance^24^.

Taken together, state math anxiety was found to vary with the paradigm in which arithmetic needed to be solved, likely due to the immediate situational demands of the task, such as the higher difficulty of producing compared to selecting an answer. Trait math anxiety moderated the paradigm effect on state math anxiety, with individuals higher in trait math anxiety showing larger differences in state math anxiety between paradigms. This pattern is consistent with theoretical models suggesting that trait anxiety influences the magnitude of state anxiety responses to situational demands^21,22,24^, pointing at the directive role of trait anxiety in generating state anxiety in specific situations. These findings have significant implications for educational settings, suggesting that the response format might be relevant in either mitigating or amplifying anxiety during mathematics for students with high levels of trait anxiety.

Complementary exploratory analyses further suggest that the heightened state math anxiety observed in production paradigms may primarily stem from fear of making mistakes rather than from increased response times. Specifically, higher levels of state math anxiety were associated with lower accuracy in production compared to decision paradigms but not with response time (Table S4). This pattern indicates that emotional tension may arise from concerns about correctness and error likelihood in open response formats rather than from time pressure.

The temporal analysis of state math anxiety adds another layer of depth to our understanding of how anxiety changes over time across different phases of mathematical tasks. State math anxiety was found to be higher before the arithmetic task, replicating results from Goetz et al.^46^, decreased during the task and finally was lower after the task, especially for individuals with high trait math anxiety. This suggests that while these individuals initially experience increased expectation anxiety, their engagement with the task may activate emotion regulation mechanisms that contribute to the reduction in state math anxiety as they progress and afterwards, they are relieved that the task is over. Alternatively, the drop in anxiety during and after the task may also reflect participants developing a more realistic perception of the actual task demands, with anticipatory anxiety inflated by generalized math fears being recalibrated once they confront the true difficulty. The findings align with research showing that activation of frontal brain regions during the anticipation phase can support cognitive control and emotion regulation, mitigating performance deficits^45^.

The multidimensional interaction model of anxiety^54^ posits that state anxiety is influenced by interactions between trait anxiety and situational factors. Individuals with higher trait anxiety may have increased state anxiety at the start of a math task compared to individuals with lower trait math anxiety. Therefore, they need to regulate their emotions and thus anxiety decreases as they become familiar with the task. Consequently, this reduction is not solely due to habituation but depends on the individual predispositions and the characteristics of the task. Our results revealed similar linear decreases in state math anxiety for individuals with higher trait math anxiety from pre to post as well as during the task. Moreover, the time-wise correlations (Table S5 and Figure S3) revealed that the association between state math anxiety and performance was strongest at the beginning of the experiment and gradually weakened over time (for response time). Thus, both (successfully) completing the task (pre-post task comparison) and habituation over time (repetitions of the arithmetic task in different paradigms) play a role in the dynamics of state math anxiety: (1) anticipation-related mechanisms, which initially drive anxiety, but become reduced as tasks are successfully performed, and (2) habituation-related mechanisms, which foster a gradual decrease in anxiety over time. Broader anxiety theory, consistent with research optimizing exposure therapy^55^, emphasizes that therapeutic gains are significantly enhanced by actively violating negative expectancies, rather than relying solely on repetition. This theoretical perspective raises interesting questions about the relative contributions of expectancy violation versus habituation in math anxiety reduction. By distinguishing these two contributing processes, our results provide new insights into how state math anxiety evolves during mathematics over time and upon task completion.

From a theoretical standpoint, the effectiveness of exposure therapy as an intervention for math anxiety might be enhanced if it incorporates strategies beyond repeated exposure to mathematical tasks^56^. The reason is that it would fail to address the critical role of anticipation-related mechanisms of anxiety. Comprehensive interventions should therefore incorporate strategies to manage pre-task expectation anxiety, such as cognitive restructuring or relaxation techniques, alongside a gradual exposure to and training in mathematics^57^. However, the relative effectiveness of different intervention components such as reducing anticipatory anxiety or facilitating habituation should be empirically tested in future studies, as research on therapeutical approaches for anxiety goes beyond the scope of the current study.

In summary, our findings underscore that math anxiety is situation-dependent (state) as well as a personal characteristic (trait), which interact over time. In individuals with high trait math anxiety, the expectation anxiety is elevated and decreases stepwise while performing mathematics. Besides, our data support the use of the state math anxiety scale^24^ as an effective tool for detecting situational differences in anxiety levels towards mathematics. Notably, similar temporal patterns were observed for state anxiety (Fig. S1), suggesting that the decrease in anxiety over time may reflect both math-specific and broader anxiety regulation processes.

The current study replicated the relationship between trait math anxiety and arithmetic performance. Consistent with the processing efficiency theory and the attentional control theory^13,14^, higher levels of anxiety were associated with slower response times^9,58,59^. Interestingly, trait math anxiety was only significantly related to response time, not accuracy, and the anxiety-complexity effect^8^ was also observed only in response time instead of accuracy (Table S2). In absence of a speed-accuracy trade-off (Table S2), this indicates that math-anxious individuals may respond more slowly without sacrificing accuracy in tasks that are in principle solvable and not too difficult (relatively high accuracy leading to ceiling effects), suggesting that anxiety is primarily associated with disruption in cognitive processing speed rather than performance quality in manageable math tasks. While this is in line with the processing efficiency theory^13,14^, stating that anxiety impairs processing efficiency (response time) but not performance effectiveness (response accuracy), other studies found the opposite pattern^60,61^. Together, these results challenge the traditional notion of a speed-accuracy trade-off in anxiety-related tasks^42^ and suggest that the relationship between math anxiety, response accuracy, and processing efficiency may vary depending on task conditions and measurement approaches.

The anxiety-complexity effect was also replicated in the current study (Table S2)^4,7,8^, with individuals with higher trait math anxiety needing particularly more time to solve complex arithmetic (involving carry or borrow operations). Therefore, math anxiety is disproportionately associated with impaired performance under more cognitively challenging conditions, because the carry and borrow operations require working memory^11^ and working memory is limited in math-anxious individuals due to intrusive thoughts^62^. These findings suggest that while math-anxious individuals may maintain accuracy through compensatory strategies, the cognitive load required for emotional regulation can extend processing time and reduce problem-solving depth in complex tasks. This supports the disruption account, which postulates that intrusive thoughts and rumination consume working memory resources, thereby impairing performance, particularly on tasks requiring high cognitive demands^9^. In turn, lower performance may induce higher state math anxiety, potentially contributing to higher trait math anxiety over time. However, this assumption requires further investigation, as state math anxiety was not assessed at the trial level in the current study.

Interestingly, trait math anxiety interacted with task difficulty (Fig. S2), but not with paradigm regarding performance. This difference highlights that trait math anxiety relates to performance in a way that is distinct from its relationship with state math anxiety. Trait math anxiety was associated with math performance dependent on task difficulty, reflecting its persistent relationship with individuals’ ability to manage increasingly complex cognitive demands. On the other hand, trait math anxiety explained the paradigm-dependent increase in state math anxiety but not the paradigm-dependent drop in performance. This suggests that trait math anxiety is associated with greater performance challenges posed by task complexity but not by task format. It should be noted that other individual differences beyond trait math anxiety, such as test anxiety, math ability, math self-concept, working memory capacity, and gender may also moderate these effects. Our relatively homogeneous and high-performing sample may have limited variability in such factors, reflecting a need for future studies.

In conclusion, trait math anxiety was associated with lower performance, especially in more complex arithmetic. Thus, the anxiety-complexity effect was replicated, with math anxiety being associated with reduced processing efficiency without a decrease in performance effectiveness.

This study offers important educational implications, emphasizing the role of task design in managing math anxiety. Integrating multiple-choice or game-based learning approaches, which have proven effective in typically developing and dyscalculic children^60,63^, can reduce cognitive load and anxiety, particularly for those with high trait math anxiety. Additionally, a repeated exposure to mathematical tasks can help reduce anxiety over time, as initial anxiety is often higher before students are familiar with the type of task they will be completing.

Future research might further investigate the mechanisms behind the interaction between math anxiety and task complexity, as well as the temporal dynamics of anxiety during more extended or varied mathematical tasks. Experimentally manipulating anticipatory anxiety would provide more direct causal evidence for the mechanisms we observed. For instance, varying pre-task instructions to increase or decrease performance expectations^44^, implementing anxiety induction procedures^64^, or testing brief anxiety-reduction interventions^65^ could help isolate the specific contribution of anticipatory mechanisms to math anxiety dynamics. Such experimental approaches would complement our correlational findings and provide stronger evidence for designing targeted interventions.

Our study is limited by several points. Regarding task complexity, the two-digit arithmetic tasks used in this study may not have been sufficiently challenging for adults, potentially contributing to the observed decrease in state anxiety as participants adapted to the task and perceived it as less difficult than expected. This habituation effect might differ if more complex math tasks were used (such as those involving fractions, larger numbers or advanced mathematics), or if time constraints were introduced (that might induce stress). Especially the task-phase decline of state math anxiety (pre-, mid-, and post-task) may be specific to tasks that are mastered well (high accuracy). Otherwise, if tasks would be more or too difficult, expectation anxiety at the beginning of the task may not decrease as much or may even increase when individuals fail most of the time. Notably, the high accuracy across paradigms indicates a potential ceiling effect, which may have reduced variability in performance and limited sensitivity to detect subtle relations with anxiety measures. Future studies could therefore consider adopting more demanding tasks to better capture individual differences in math performance.

For sample diversity, the relatively small and homogenous adult sample may limit the variance in our study to interindividual differences and the generalizability of the results, particularly to developmental stages. Specifically, our university student sample showed relatively high math competence (as evidenced by high accuracy rate), limiting variability in math ability. Additionally, the unbalanced gender distribution (71% female) should be noted, as females typically report higher math anxiety^66^. We also did not assess other potentially relevant individual differences such as working memory capacity. Future research should include a more diverse population in terms of age, educational background, and cultural context, as well as standardized measures of math ability to disentangle the effects of math ability from math anxiety, to validate the findings across different groups. Neurocognitive approaches would facilitate the development of more effective, targeted interventions to reduce math anxiety and improve learning outcomes for both typically developing and math-disabled students^63^.

As for feedback mechanisms, this study did not incorporate feedback for each arithmetic problem. Research shows that feedback can have complex effects on emotions and learning outcomes, while some studies indicate that feedback, especially corrective feedback, can evoke negative emotions and influence learning outcomes^67^, other research suggests that feedback may reduce math anxiety, particularly in higher education settings^68^. Incorporating different types of feedback in future studies would provide a deeper understanding of how feedback impacts state math anxiety and performance.

With respect to self-reported measures, relying solely on self-reported measures of math anxiety may introduce bias. For instance, the fact that women report more anxiety in self-reports than men have been attributed to a response bias with men feeling more uncomfortable to admit anxiety^69^. Moreover, there is a discrepancy between state and trait math anxiety with girls reporting more trait but not state math anxiety than boys^34^, and autistic boys reporting more state but not trait math anxiety than non-autistic boys^70^. Future research should adopt multidimensional assessments, combining behavioral experiments and self-report questionnaires with psychophysiological markers and neuroimaging data to obtain more objective and comprehensive data^24,71^.

To conclude, this study reveals the dynamic nature of state math anxiety in arithmetic tasks, focusing on the dependency on trait math anxiety, the effects of paradigms, and temporal trends. The results show that production paradigms, which require generating answers, increase state math anxiety compared to decision paradigms, especially in individuals with higher trait math anxiety. Over time, state math anxiety decreases linearly from pre- to post-arithmetic tasks and during repetitions of the task. The anxiety-complexity effect was observed, with higher trait math anxiety being associated with worse performance particularly in complex arithmetic. These findings offer valuable insights for designing educational approaches that reduce anxiety and enhance learning outcomes.

## Methods

### Participants

The sample included 65 adults (17 male, 46 female, 2 diverse; age: *M* = 22.86 years, *SD* = 3.80 years, *Range* = 19–37 years), taken from the study on paradigms^40^. Among all subjects, 57 were right-handed and 8 were left-handed. Inclusion criteria for participants were an age between 18 and 40 years, native German speakers, and no dyscalculia or other learning disorders (e.g., attention deficit hyperactivity disorder). For participation, all subjects received student credits or monetary reimbursement. Informed written consent was obtained from all subjects and the study was conducted following the latest version of the Declaration of Helsinki.

## Material

To assess trait and state (math) anxiety, we administered the following questionnaires. The Abbreviated Math Anxiety Scale (AMAS)^72^ was used to measure trait math anxiety. The questionnaire consists of 9 items with a 5-point Likert scale, ranging from 1 (low anxiety) to 5 (high anxiety). The scale demonstrated strong internal consistency (Cronbach’s *α* = 0.90), good test-retest reliability (*r* = 0.85), and good convergent and divergent validity^72^. In the present study, a German translated version was used, which also showed very good internal reliability (Cronbach’s *α* = 0.89 and ordinal *α* = 0.94).

The State Math Anxiety Scale (SMA)^24^ was used to measure state math anxiety. The questionnaire consists of 7 items that capture the emotional, cognitive, and physiological aspects of math anxiety and 2 control items for enjoyment and boredom, with a 5-point Likert scale ranging from 1 (not at all) to 5 (very much). The German scale demonstrated strong internal consistency (Cronbach’s *α* > .90, ranging from 0.91 to 0.95) and good validity^24^. As the SMA is situational with questions relating to the math task at hand, some questions would be inappropriate if not presented during the context of a math task (before and after the math task). Therefore, we only kept one item of the SMA (SMA-1) to measure state math anxiety before and after the math task: “How math-anxious do you feel right now?” (adapted from SIMA^73^). In the present study, the SMA also showed good internal consistency (Cronbach’s α ≥ 0.80 during the arithmetic task, with ordinal α ranging from 0.70 to 0.90).

The short German version of the State-Trait Anxiety Inventory (STAI-SKD)^74^ was used to measure state anxiety. The questionnaire consists of 5 items reflecting the current emotional state, which should be rated on a 5-point Likert scale ranging from 1 (not at all) to 4 (very much). The internal consistency of the STAI-SKD was satisfactory (Cronbach’s *α* = 0.76)^74^. In the present study, the STAI-SKD also demonstrated good internal consistency (Cronbach’s α and ordinal *α* ≥ 0.80 at each measurement time point). Both state math anxiety and state anxiety were measured to distinguish math-specific emotional responses from broader anxiety dynamics during task performance; however, the constructs of state math anxiety and state anxiety in the math context can be considered the same^24^.

All questionnaires were administered using a paper-and-pencil format.

The arithmetic task followed the same procedure as described in our previous study^24^. Each arithmetic problem consisted of two two-digit operands that resulted in a two-digit solution. In a 2 × 2 design, the problems included addition with (e.g., 36 + 27) or without (e.g., 32 + 24) carrying (a carry operation in addition is required when the sum of the units of the operands exceeds 9, with a decade to be carried over) and subtraction with (e.g., 63–25) or without (e.g., 69–23) borrowing (a borrow operation in subtraction is required whenever the unit of the subtrahend is larger than the unit of the minuend, and hence a decade has to be borrowed). Each of these four conditions consisted of 24 arithmetic problems, resulting in 96 problems within one stimulus set. Six stimulus sets were created^24^ and were matched in the numerical magnitude of the operands and the result, overall problem size, and counterbalanced in the position of the larger operand. The stimulus sets did not entail trivial cases such as pure decades (e.g., 20), ties (e.g., 22), or unit/decade repetitions. Subtraction problems were constructed as inverse addition problems. Note that the stimulus sets were matched, but not identical to avoid trial-specific learning across paradigms. The distractor in decision paradigms differed from the target at the unit position ( ± 2) or at the decade position ( ± 10). The dependent variables for arithmetic performance are accuracy (ACC) and response time (RT).

**Fig. 3:**
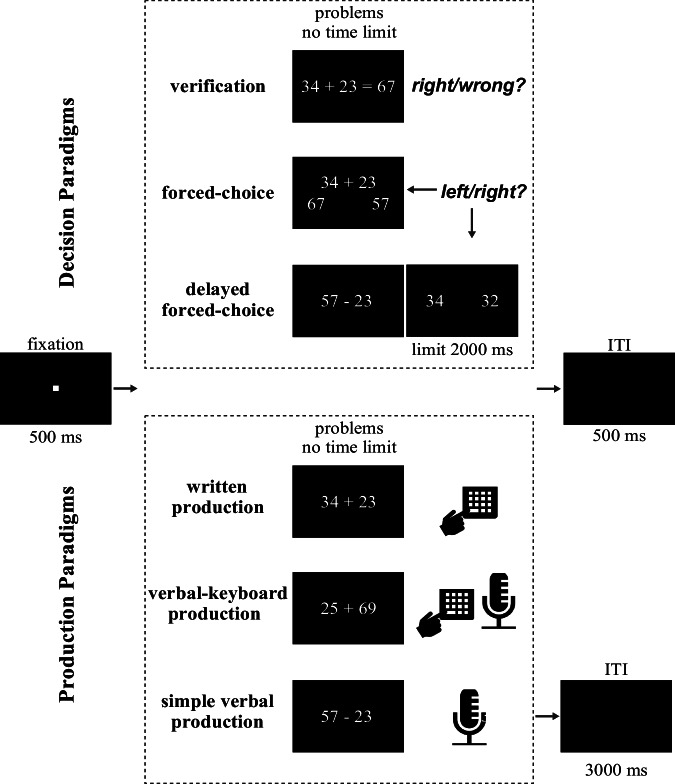
Different arithmetic paradigms. Each trial began with a 500 ms fixation, followed by the presentation of an arithmetic problem. In decision paradigms (verification, forced-choice, delayed forced-choice), participants judged the correctness of a presented solution or selected the correct answer from two alternatives. The delayed forced-choice paradigm additionally required participants to indicate by pressing the spacebar on the keyboard when they had mentally calculated the answer before response options appeared (time limit was 2000 ms). In production paradigms (written production, verbal-keyboard production, simple verbal production), participants generated the answer either by typing or verbalizing it. An inter-trial interval (ITI) of 500 ms was used for all paradigms except the simple verbal production paradigm, which used 3000 ms. No time limit was imposed for problem solving, except for the response in the delayed forced-choice condition.

Two types of paradigms were employed in this study: decision vs. production paradigms (see Fig. 3). In decision paradigms (verification, forced-choice, delayed forced-choice), participants should decide whether the given answer was correct or select the correct answer. In production paradigms (written production, verbal-keyboard production, simple verbal production), participants should calculate the answer and type it or say it aloud. The six specific paradigms used were the following (for a detailed description of the paradigms see Yao et al.^24^): In the verification paradigm, participants need to indicate whether the given answer is right or wrong. In the forced-choice paradigm, participants need to choose one out of two given answer options presented simultaneously with the arithmetic problem. In the delayed forced-choice paradigm, first the arithmetic problem was shown until participants pressed the space bar indicating that they had calculated the answer in mind; afterwards, the two answer options (target and distractor) appeared from which participants chose the calculated answer (with a time limit of 2000 ms). In the written production paradigm, participants need to type the answer directly in a number keyboard. In the verbal-keyboard production paradigm, participants need to verbalize the answer while pressing a button on the keyboard (to record response time). In the simple verbal production paradigm, participants need to verbalize the answer directly (while response time is recorded by voice key).

### Procedure

**Fig. 4:**
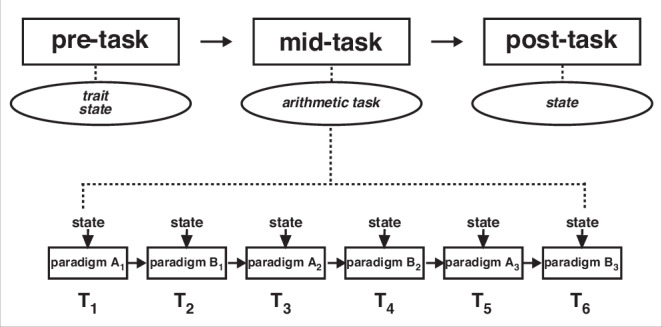
Flowchart of the study procedure. T_1_ to T_6_ indicate the six measurement time points during the arithmetic task. Trait math anxiety was measured pre-task, state math anxiety with one item and state anxiety were measured pre-task and post-task. State (math) anxiety was measured during the break of each paradigm after half of the arithmetic problems. Paradigm A and B refer to the two paradigm types (production vs. decision) presented in alternating order. Subcategories A1, A2, A3 and B1, B2, B3 further distinguish the three production paradigms and the three decision paradigms. The alternating order of paradigms was counterbalanced across participants.

The procedure started with questionnaires on trait math anxiety (AMAS), state math anxiety (SMA-1), and state anxiety (STAI-SKD). Then the arithmetic task was conducted computer-based using the OpenSesame 3.3.10^76^. Each of the 3 decision and 3 production paradigms were presented in alternating blocks (see Fig. 4), with the order of paradigm blocks following a Latin Square design^40^, i.e., participants were evenly assigned to six counterbalanced sequences in which each paradigm appears once in each ordinal position. This controls for position effects, though it does not completely counterbalance all possible orders. Each participant completed all six paradigms (one stimulus set of 96 problems per paradigm), resulting in a total of 576 arithmetic problems per participant. The arithmetic problems within each block were presented in randomized order. The participants were instructed to solve the arithmetic problems as quickly and accurately as possible. Within each paradigm block, there was a break after half of the arithmetic problems, in which participants were asked to fill in the questionnaires on state math anxiety (SMA) and state anxiety (STAI-SKD), resulting in 6 measurements during the arithmetic task. After the arithmetic task, state math anxiety (SMA-1) and state anxiety (STAI-SKD) were assessed again (for a flowchart of the experiment see Fig. 4). The whole experiment lasted ~2 h.

## Data analysis

Data exclusion criteria were preregistered and applied as follows (for details see Yao et al.^75^, Table S1): Participants were case-wise removed with missing data in the arithmetic task, with an ACC below 50% per production paradigm or below 75% per decision paradigm (due to a 50% chance level), or with a mean RT >3 median absolute deviation (*MAD*)^76^ above or below the group *Median* for the respective paradigm. According to these criteria, the final sample size ranged from 59−65 participants across paradigms (verification: *N* = 59; forced-choice: *N* = 59; delayed forced-choice: *N* = 63; written production: *N* = 63; verbal-keyboard production: *N* = 65; simple verbal production: *N* = 64). In the arithmetic task, trials were removed from RT analysis according to the following criteria: false equation trials in the verification paradigm (also for ACC), incorrectly solved trials (i.e., errors and missing; 8%), RTs below 200 ms (anticipations; 0%), RTs >3 *MAD* above or below the individual *Median* for the respective paradigm (outliers; 6%), or distance between first and second RT >3 *MAD* above or below the individual *Median* for the respective paradigm (delayed forced-choice, written production, and verbal-keyboard production; 0%).

Trial-level accuracy data (0/1) were analyzed using generalized linear mixed-effects models (GLMMs) with a binomial distribution and logit link function^77^. For analyses at the condition- or subject-level (speed–accuracy LMM analysis and exploratory correlation analysis in the current study), logit-transformed accuracy data were used to stabilize variance and approximate a normal distribution. No transformation was applied to RT data, as their distributions were sufficiently normalized following the MAD-based outlier removal procedure.

In the questionnaires, missing data were handled by calculating mean instead of sum scores, with higher values indicating higher anxiety. Missing data were handled by calculating mean instead of sum scores, with higher values indicating higher anxiety. No more than one item was missing per questionnaire and participant. For data analysis, we centered continuous predictors, including trait math anxiety (AMAS), response time (for speed-accuracy trade-off analysis), and time (3 time points and 6 time points) data to facilitate the interpretation of their effects on the dependent variable and to improve the stability of the statistical model^78^. To center the variables, we subtracted the mean value of the variable in our sample from the mean value of each participant so that the centered score represents the deviation of the individual value from the overall sample.

The preregistered data analysis was conducted using the statistical computing software R^79^, including the R packages tidyr^80^, dplyr^81^, ggplot2^82^, afex^83^, and lmerTest^84^. Given the hierarchical nature of the data, with multiple observations nested within participants, we used Linear Mixed Models (LMMs) and Generalized Linear Mixed Models (GLMMs) to analyze the data. This approach is widely established in cognitive and arithmetic research because it accounts for both between- and within-participant variability and avoids the independence assumptions inherent in traditional ANOVAs^85,86^. All Linear Mixed Models (LMM) and Generalized Linear Mixed Models (GLMM) were fitted with the function lmer from the lmerTest R package, with maximum-likelihood estimation for the fixed effects and logit as link function for the GLMM. As preregistered, we conducted model selection using a top-down strategy^87^ based on the ANOVA, which provides a principled way to balance model fit and parsimony. We first fitted a full model, then sequentially reduced the random effect structure, followed by reducing the fixed effect structure, and finally reported the final model. Detailed model specifications and all selection steps are reported in Table S3.

## Supplementary information

Supplementary materials

## Data Availability

The materials, data, and analysis code (R scripts) for this study are openly available on the Open Science Framework (OSF) at https://osf.io/z8cqm/overview.

## **Code availability**

The R code for analysis for each hypothesis are available in the OSF repository (https://osf.io/z8cqm/overview).
