# Supplementary material for: The dynamics of state math anxiety vary by paradigm and timing during arithmetic
Source: NPJ Sci Learn. 2026 Jan 20;11:10. doi: 10.1038/s41539-025-00398-z (PMC12847727; doi:10.1038/s41539-025-00398-z)
Supplement: Supplementary file 1 — Supplementary materials [file 41539_2025_398_MOESM1_ESM.pdf]

Supplementary Materials

Title: The dynamics of state math anxiety vary by paradigm and timing during arithmetic

Table S1. Arithmetic performance and mid-task state anxiety across paradigms

	**Decision paradigms**			**Production paradigms**		
	verification	forced-choice	delayed forced-choice	written production	verbal-keyboard production	simple verbal production
Arithmetic performance						
Accuracy						
addition simple	0.93 (0.08)	0.96 (0.04)	0.95 (0.06)	0.92 (0.07)	0.96 (0.04)	0.92 (0.08)
addition complex	0.94 (0.06)	0.93 (0.08)	0.92 (0.08)	0.90 (0.08)	0.91 (0.08)	0.88 (0.11)
subtraction simple	0.94 (0.07)	0.95 (0.05)	0.94 (0.05)	0.92 (0.06)	0.92 (0.07)	0.90 (0.10)
subtraction complex	0.91 (0.08)	0.92 (0.07)	0.91 (0.08)	0.87 (0.12)	0.88 (0.10)	0.82 (0.15)
Reaction time						
addition simple	3.20 (0.84)	3.04 (0.64)	2.96 (0.75)	3.86 (0.81)	3.86 (0.81)	3.02 (0.80)
addition complex	4.35 (1.01)	3.87 (0.86)	4.21 (1.17)	5.30 (1.32)	5.30 (1.32)	4.27 (1.03)
subtraction simple	4.03 (1.09)	3.64 (0.96)	3.73 (1.01)	4.69 (1.15)	4.69 (1.15)	3.81 (1.00)
subtraction complex	5.02 (1.23)	4.50 (1.02)	4.84 (1.23)	6.04 (1.33)	6.04 (1.33)	5.01 (1.19)
Questionnaires						
mid-task state math anxiety	1.59 (0.72)	1.54 (0.63)	1.58 (0.70)	1.62 (0.73)	1.82 (0.83)	2.19 (1.00)
mid-task state anxiety	1.55 (0.69)	1.41 (0.50)	1.41 (0.61)	1.43 (0.57)	1.78 (0.76)	2.12 (0.95)

Notes. Values are presented as *M* (*SD*) for each decision and production paradigm. State (math) anxiety was measured during the break at the midpoint of each paradigm of the arithmetic task.

Part 1: Additional analysis and results

Table S2. LMM/GLMM results

**Predictors**	***β***	***CI***	***t / z***	***p***	***R²***
**Model S1**: LMM for three-time points analysis on state anxiety					.62
(intercept)	1.57	1.46 – 1.69	27.20	**< .001**	
time	-0.27	-0.34 – -0.20	-7.46	**< .001**	
trait math anxiety	0.31	0.14 – 0.48	3.64	**< .001**	
time × trait math anxiety	-0.20	-0.31 – -0.10	-3.86	**< .001**	
time²					
time² × trait math anxiety					
**Model S2**: LMM for six-time points analysis on state anxiety					.71
(intercept)	1.62	1.36 – 1.89	12.08	**< .001**	
time	-0.09	-0.11 – -0.07	-7.59	**< .001**	
time²	-0.00	-0.02 – 0.01	-0.23	.820	
trait math anxiety	0.18	-0.03 – 0.39	1.67	.096	
time² × trait math anxiety	0.03	0.00 – 0.05	2.10	**.036**	
time × trait math anxiety					
**Model S3**: LMM for trait math anxiety and difficulty on response time					.39
(intercept)	3.38	3.18 – 3.59	32.30	**< .001**	
trait math anxiety	0.30	0.00 – 0.59	1.97	**.049**	
difficulty	1.20	1.13 – 1.28	30.70	**< .001**	
trait math anxiety × difficulty	0.15	0.09 – 0.20	5.46	**< .001**	
**Model S4**: GLMM for trait math anxiety and difficulty on accuracy					.14
(intercept)	2.83	2.66 – 3.00	32.07	**< .001**	
difficulty	-0.47	-0.57 – -0.38	-9.76	**< .001**	
trait math anxiety					
trait math anxiety × difficulty					
**Model S5**: GLMM for speed-accuracy trade-off					.68
(intercept)	2.54	2.42 – 2.66	42.31	**< .001**	
paradigm [production]	-0.29	-0.39 – -0.19	-5.77	**< .001**	
trait math anxiety					
response time					
paradigm × trait math anxiety					
paradigm × response time					
trait math anxiety × response time					
paradigm × trait math anxiety × response time					

Notes. *t* value for LMM and *z* value for GLMM. Time and trait math anxiety were centered. Difficulty was dummy coded with simple as reference for complex. Conditional R^2 quantifies the proportion of variance explained by the entire model, including both fixed and random effects.

Time analysis for state anxiety pre-, mid-, and post-arithmetic task

Similar to the LMM results for state math anxiety (see Model D in Table 2), the final LMM for state anxiety (see Model S1 in Table S2, Figures S1a & S1b) included fixed effects for time, trait math

anxiety, and the interaction between time and trait math anxiety, with a random intercept for subject. The main effect of time indicates that state anxiety decreases over time, with an estimated reduction of -0.27 per time point. The main effect of trait math anxiety shows that for each unit increase in trait math anxiety, state anxiety increases by an estimated 0.31, suggesting that individuals with higher levels of trait math anxiety exhibit higher levels of state anxiety. Additionally, the interaction effect between time and trait math anxiety, with an estimate of -0.20, implies that the decrease in state anxiety over time is more pronounced for individuals with higher trait math anxiety.

Time analysis for state anxiety during the arithmetic task

Different from the LMM results for state math anxiety (see Model E in Table 2), the final LMM for state anxiety (see Model S2 in Table S2, Figures S1c & S1d) included, besides the fixed effect for time, fixed effects for time^2, trait math anxiety, and the interaction between time^2 and trait math anxiety, with random intercepts for subject and paradigm. The main effect of time indicates that state anxiety decreases over time, with an estimated reduction of -0.09 per time point. Different to the larger linear decrease of state math anxiety across six measurement times during arithmetic task for individuals with higher trait math anxiety, the interaction between time^2 and trait math anxiety, with an estimate of 0.03, indicates a non-linear relationship: individuals with higher trait math anxiety show an particularly an initial decrease in state anxiety that stabilizes during the task, reflecting an adaptive or reactive response to the task demands.

Figure S1. State anxiety changes across time

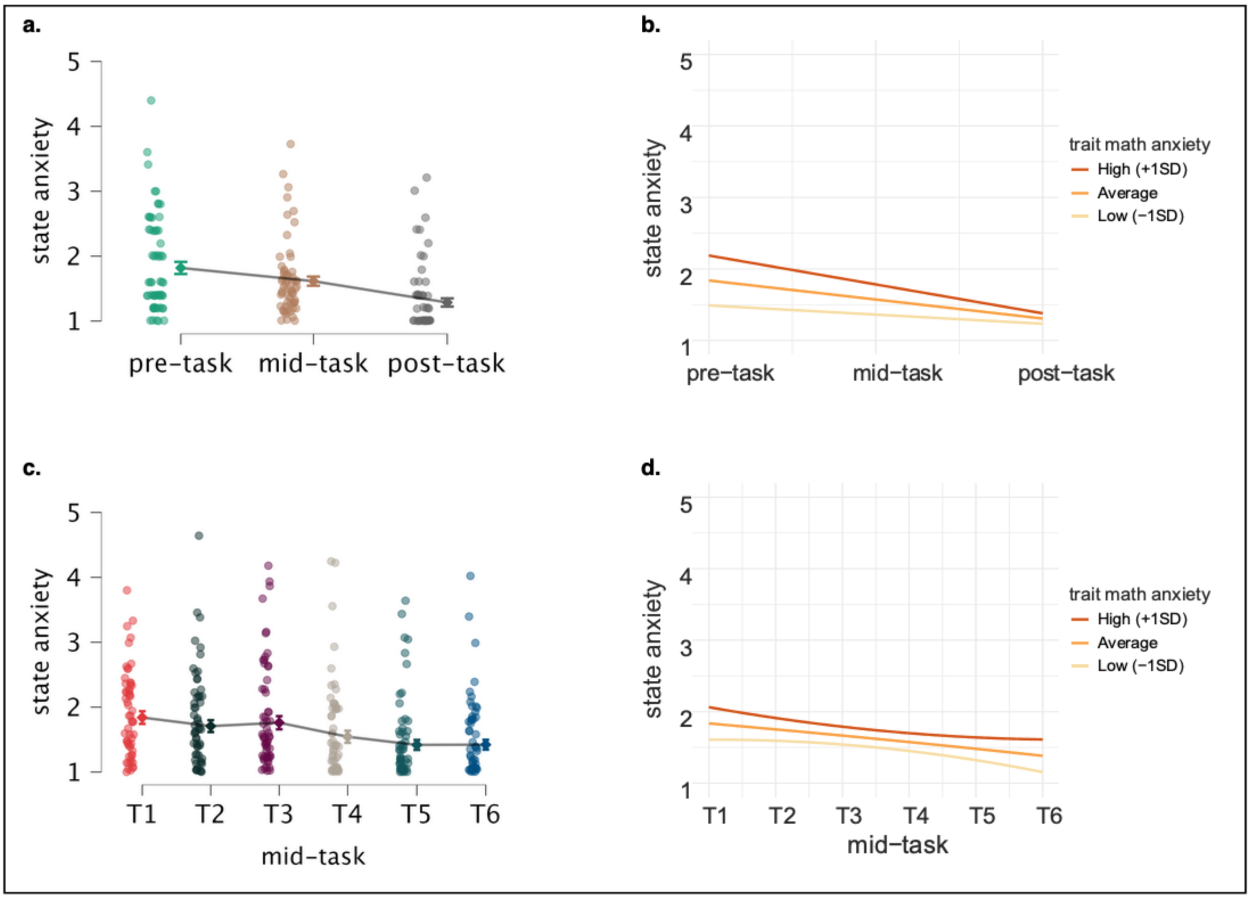

Notes. (a) and (c) show the decrease in state anxiety across the three task phases and across the six

measurement times during the arithmetic task, respectively. Error bars represent the standard error of the mean (*SEM*). (b) and (d) show a simple slope analysis for state anxiety depending on trait math anxiety (average level, high level with 1 *SD* above the average, and low level with 1 *SD* below the average) across the three task phases and during the arithmetic task.

Anxiety-complexity effect

An LMM with RT as dependent variable and a GLMM with ACC as dependent variable were conducted including fixed effects for trait math anxiety, difficulty (complex vs. simple), and their interaction. The (G)LMMs further included random intercepts for both subject and item, as well as – not preregistered – a random slope for paradigm, because we found significant differences in performance between paradigms (Yao et al., 2025).

The final LMM for RT (see Model S3 in Table S2, Figure S2) included fixed effects for trait math anxiety, difficulty and their interaction, with random intercepts for subject and item as well as a random slope for paradigm. The main effect of trait math anxiety indicates that for every unit increase in trait math anxiety, the response time increases by an estimate of 0.30 s, so that individuals with higher trait math anxiety take longer to solve arithmetic. The main effect of difficulty indicates that complex arithmetic (with carry/borrow) takes longer to be solved by an estimate of 1.20 s than simple arithmetic (without carry/borrow). The interaction of trait math anxiety and difficulty indicates an anxiety-complexity effect, so that with increasing trait math anxiety the difficulty effect increases by an estimate of 0.15 s.

Different from RT, the final GLMM for ACC (see Model S4 in Table S2) included only a fixed effect for difficulty, with random intercepts for subject and item as well as a random slope for paradigm. The main effect of difficulty indicates that accuracy is higher by an estimate of -0.47 for simple arithmetic (without carry/borrow) compared to complex arithmetic (with carry/borrow).

Together, the results suggest that the difficulty effect was replicated in both RT and ACC. For RT, further an anxiety-complexity effect was found, so that individuals with higher trait math anxiety needed longer for arithmetic than individuals with lower math anxiety, especially for more complex arithmetic.

Speed-accuracy trade-off

An LMM with ACC as the dependent variable was conducted (but not a GLMM – as preregistered – because when including response time as a fixed factor, the model failed to converge on a trial level; instead, we performed an LMM on a subject level with average accuracy per participant as dependent variable) including fixed effects for paradigm, RT, trait math anxiety, and their interactions. The LMM further included a random intercept for subject (but not random slopes for RT and paradigm as preregistered, because incorporating this would have made the random effects structure too complex given the limited number of observations).

The final LMM on ACC (see Model S5 in Table S2) only included a fixed effect for paradigm – but not response time. Therefore, the results did not reveal a speed-accuracy trade-off for the current study.

Figure S2. The anxiety-complexity effect

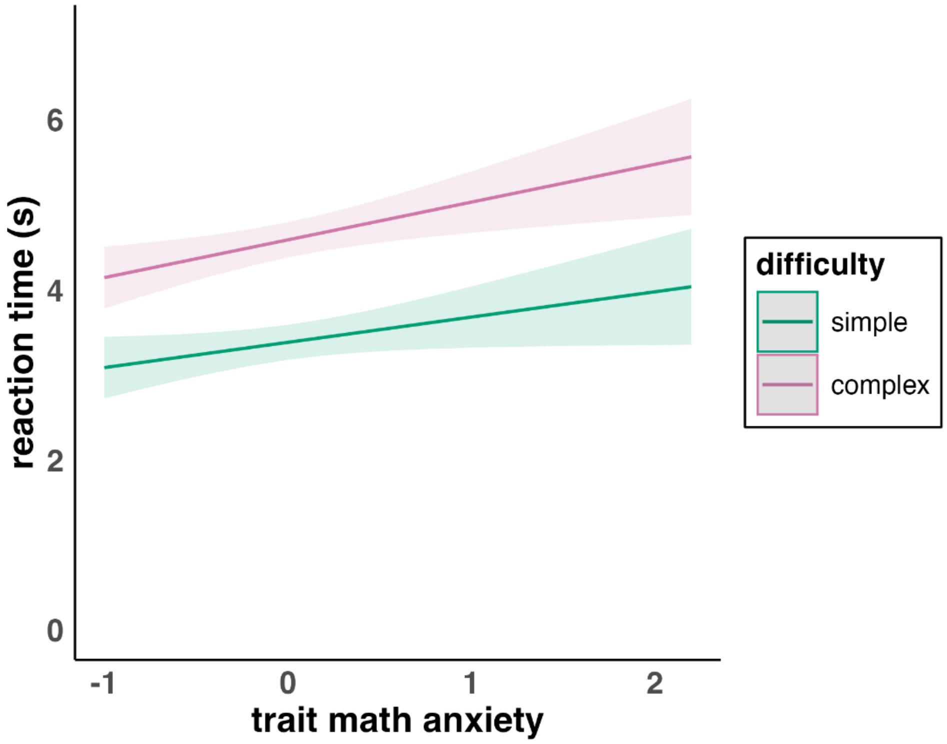

Notes: The anxiety-complexity is indicated by the interaction of trait math anxiety and difficulty on arithmetic performance in terms of response times. Individuals with higher trait math anxiety show higher carry/borrow effects than individuals with lower trait math anxiety, i.e., they need even more time for complex arithmetic (with carry/borrow operation) as compared to simple arithmetic (without carry/borrow operation).

Part 2: Model selection process

Table S3. Full models

Predictors	*β*	*CI*	*t / z*	*p*	R^2
**Model A**: LMM for paradigm and trait math anxiety on state math anxiety					.87
(intercept)	1.57	1.42 – 1.72	20.74	**< .001**	
paradigm	0.29	0.21 – 0.38	6.64	**< .001**	
trait math anxiety	0.28	0.06 – 0.50	2.52	**.013**	
paradigm × trait math anxiety	0.24	0.11 – 0.37	3.64	**< .001**	
**Model B**: LMM for trait math anxiety and paradigm on response time					.42
(intercept)	3.96	3.75 – 4.16	37.47	**< .001**	
paradigm	0.41	0.30 – 0.52	7.48	**< .001**	
trait math anxiety	0.36	0.06 – 0.65	2.39	**.017**	
paradigm × trait math anxiety	*0.10*	*0.06 – 0.26*	*1.21*	.226	
**Model C**: GLMM for trait math anxiety and paradigm on accuracy					.15
(intercept)	3.18	2.74 – 3.62	14.06	**< .001**	
paradigm	-0.58	-0.94 – -0.22	-3.16	**.002**	
trait math anxiety	-0.25	-0.46 – 0.04	-2.37	**.018**	
paradigm × trait math anxiety	*0.15*	*-0.02 – 0.32*	*1.74*	*.081*	
**Model D**: LMM for three-time points analysis on state math anxiety					.70
(intercept)	1.66	1.49 – 1.83	19.23	**< .001**	
time	-0.31	-0.39 – -0.22	-7.39	**< .001**	
trait math anxiety	0.51	0.26 – 0.77	4.03	**< .001**	
time × trait math anxiety	-0.31	-0.43 – -0.19	-5.10	**< .001**	
time^2	*0.11*	*0.03 – 0.25*	*1.52*	*.131*	
time^2 *× trait math anxiety*	*0.13*	*0.08 – 0.34*	*1.24*	*.216*	
**Model E**: LMM results for six-time points analysis on state math anxiety					.75
(intercept)	1.71	1.47 – 1.96	13.68	**< .001**	
time	-0.11	-0.13 – -0.08	-8.95	**< .001**	
trait math anxiety	0.37	0.14 – 0.60	3.22	**.001**	
time × trait math anxiety	-0.07	-0.11 – -0.04	-3.95	**< .001**	
time^2	*0.00*	*0.01 – 0.02*	*0.42*	*.677*	

time^2 *× trait math anxiety*	*0.01*	*0.01 – 0.03*	*0.79*	*.431*	
**Model S1**: LMM for three-time points analysis on state anxiety					0.62
(intercept)	1.61	1.47 – 1.75	22.73	**< .001**	
time	-0.27	-0.34 – -0.20	-7.45	**< .001**	
trait math anxiety	0.17	0.18 – 0.51	0.97	.335	
time × trait math anxiety	-0.37	0.75 – 0.00	-1.97	.051	
time^2	*-0.37*	*0.75 – 0.00*	*-1.97*	*.051*	
time^2 *× trait math anxiety*	*0.09*	*0.10 – 0.27*	*0.93*	*.352*	
**Model S2**: LMM for six-time points analysis on state anxiety					.71
(intercept)	1.62	1.36 – 1.89	12.08	**< .001**	
time	-0.09	-0.11 – -0.07	-7.60	**< .001**	
time^2	-0.00	-0.02 – 0.01	-0.23	.819	
trait math anxiety	0.18	-0.03 – 0.39	1.67	.096	
time^2 × trait math anxiety	0.03	0.00 – 0.05	2.11	**.036**	
time × trait math anxiety	*-0.02*	*-0.06 – 0.01*	*-1.37*	*.171*	
**Model S3**: LMM for trait math anxiety and difficulty on response time					.39
(intercept)	3.38	3.18 – 3.59	32.30	**< .001**	
trait math anxiety	0.30	0.00 – 0.59	1.97	**.049**	
difficulty	1.20	1.13 – 1.28	30.70	**< .001**	
trait math anxiety × difficulty	0.15	0.09 – 0.20	5.46	**< .001**	
**Model S4**: GLMM for trait math anxiety and difficulty on accuracy					.13
(intercept)	17.22	14.57 – 20.35	33.40	**< .001**	
difficulty	0.62	0.57 – 0.69	-9.72	**< .001**	
trait math anxiety	*0.82*	*0.66 – 1.02*	*-1.80*	*.071*	
trait math anxiety × difficulty	*0.97*	*0.87 – 1.08*	*-0.61*	*.542*	
**Model S5**: GLMM for speed-accuracy trade-off					.67
(intercept)	2.54	2.42 – 2.66	42.31	**< .001**	
paradigm	-0.29	-0.39 – -0.19	-5.77	**< .001**	
trait math anxiety	-0.64	-1.38 – 0.99	-1.73	.086	
response time	-0.02	-0.16 – 0.13	-0.26	.798	
paradigm × trait math anxiety	0.26	-0.51 – 1.04	0.68	.501	
paradigm × response time	-0.07	-0.20 – 0.07	-1.00	.322	

trait math anxiety × response time	0.11	-0.06 – 0.29	1.30	.197
paradigm × trait math anxiety× response time	-0.05	-0.22 – 0.13	-0.53	.595

Notes. *t* value for LMM and *z* value for GLMM for full models. Factors in italics are removed factors compared with reduced final model. Time and trait math anxiety were centered. Difficulty was dummy coded with simple as reference for complex. Conditional R^2 quantifies the proportion of variance explained by the entire model, including both fixed and random effects.

1. Paradigm-dependent analysis for state math anxiety

Full model	*state math anxiety ~ paradigm + trait math anxiety + paradigm : trait math anxiety + (1 | subject)*	
Step 1	*state math anxiety ~ paradigm + trait math anxiety + (1 | subject)*	The model excluding the interaction between paradigm and trait math anxiety was significantly different from the original model (χ^2 = 12.02, $p < .001$). Therefore, we retained the interaction in the final model.
Final Model	**Model A**: *state math anxiety ~ paradigm + trait math anxiety + paradigm : trait math anxiety + (1 | subject)*	Final model = Full model
Random effects	subject (intercept, variance = 0.31, SD = 0.56) (residual, variance = 0.06, SD = 0.25)	

2. Trait math anxiety and paradigm effects on arithmetic performance

(1) LMM for response time:

Full model	*response time ~ trait math anxiety + paradigm + trait math anxiety : paradigm + (1 + paradigm | subject) + (1 | item)*	
Step 1	*response time ~ trait math anxiety + paradigm + trait math anxiety : paradigm + (1 | subject) + (1 | item)*	The model excluding the random slope for paradigm was significantly different from the original model (χ^2 = 334.03, $p < .001$). Therefore, we retained the random slope for paradigm in the final model.
Step 2	*response time ~ trait math anxiety + paradigm + trait math anxiety : paradigm + (1 + paradigm | subject)*	The model excluding the random intercept for item was significantly different from the original model (χ^2 = 4808.20, $p < .001$). Therefore, we retained the random intercept for item in the final model.
Step 3	*response time ~ trait math anxiety + paradigm + (1 + paradigm | subject) + (1 | item)*	The model excluding the interaction of trait math anxiety and paradigm was not significantly different from the original

		model (χ^2 = 1.48, *p* = .223). Therefore, we removed the interaction of trait math anxiety and paradigm in the final model.
Step 4	*response time ~ trait math anxiety + (1 + paradigm | subject) + (1 | item)*	The model excluding the fixed effect of paradigm was significantly different from the original model (χ^2 = 42.55, *p* < .001). Therefore, we retained the fixed effect of paradigm in the final model.
Step 5	*response time ~ paradigm + (1 + paradigm | subject) + (1 | item)*	The model excluding the fixed effect of trait math anxiety was significantly different from the original model (χ^2 = 6.92, *p* = .031). Therefore, we retained the fixed effect of trait math anxiety in the final model.
Final model	**Model B**: *response time ~ trait math anxiety + paradigm + (1 + paradigm | subject) + (1 | item)*	Final model = Step 3
Random effects	item (intercept, variance = 0.66, SD = 0.81)<br>subject (intercept, variance = 0.65, SD = 0.80)<br>paradigm (slope, variance = 0.17, SD = 0.41, *r* = 0.07)<br>(residual, variance = 2.10, SD = 1.45)	

(2) GLMM for accuracy:

Full model	*accuracy ~ trait math anxiety + paradigm + trait math anxiety : paradigm + (1 + paradigm | subject) + (1 | item)*	
Step 1	*accuracy ~ trait math anxiety + paradigm + trait math anxiety : paradigm + (1| subject) + (1 | item)*	The model excluding the random slope for paradigm was significantly different from the original model (χ^2 = 29.18, *p* < .001). Therefore, we retained the random slope for paradigm in the final model.
Step 2	*accuracy ~ trait math anxiety + paradigm + trait math anxiety : paradigm + (1 + paradigm | subject)*	The model excluding the random intercept for item was significantly different from the original model (χ^2 = 158.88, *p* < .001). Therefore, we retained the random intercept for item in the final model.
Step 3	*accuracy ~ trait math anxiety + paradigm + (1 + paradigm | subject) + (1 | item)*	The model excluding the interaction of trait math anxiety and paradigm was not significantly different from the original model (χ^2 = 2.89, *p* = .089). Therefore, we removed the interaction of trait math anxiety and paradigm in the final

		model.
Step 4	*accuracy ~ trait math anxiety + (1 + paradigm | subject) + (1 | item)*	The model excluding the fixed effect of paradigm item was significantly different from the original model (χ^2 = 19.61, $p < .001$). Therefore, we retained the fixed effect of paradigm in the final model.
Step 5	*accuracy ~ trait math anxiety + paradigm + (1 + paradigm | subject) + (1 | item)*	The model excluding the fixed effect of trait math anxiety was significantly different from the original model (χ^2 = 6.50, $p = .039$). Therefore, we retained the fixed effect of trait math anxiety in the final model.
Final model	**Model C**: *response time ~ trait math anxiety + paradigm + (1 + paradigm | subject) + (1 | item)*	Final model = Step 3
Random effects	item (intercept, variance = 0.21, SD = 0.46)<br>subject (intercept, variance = 0.28, SD = 0.53)<br>paradigm (slope, variance = 0.13, SD = 0.37, r = -0.07)	

3. State (math) anxiety across three-time points

(1) LMM for state math anxiety across three-time points:

Full model	*state math anxiety ~ time + time^2 + trait math anxiety + time : trait math anxiety + time^2 : trait math anxiety + (1 | subject)*	
Step 1	*state math anxiety ~ time + time^2 + trait math anxiety + time^2 : trait math anxiety + (1 | subject)*	The model excluding the interaction of time and trait math anxiety was significantly different from the original model (χ^2 = 24.40, $p < .001$). Therefore, we retained the interaction of time and trait math anxiety in the final model.
Step 2	*state math anxiety ~ time + time^2 + trait math anxiety + time : trait math anxiety + (1 | subject)*	The model excluding the interaction of $time^2$ and trait math anxiety was not significantly different from the original model (χ^2 = 1.58, $p = .209$). Therefore, we removed the interaction of $time^2$ and trait math anxiety in the final model.
Step 3	*state math anxiety ~ time + trait math anxiety + time : trait math anxiety + (1 | subject)*	The model excluding the fixed effect of $time^2$ was not significantly different from the original model (χ^2 = 3.90, p = .142). Therefore, we removed the fixed effect of $time^2$ in the final model.

Final Model	**Model D**: *state math anxiety ~ time + trait math anxiety + time : trait math anxiety + (1 | subject)*	Final model = Step 3
Random effects	subject (intercept, variance = 0.26, SD = 0.51) (residual, variance = 0.22, SD = 0.47)	

(2) LMM for state anxiety across three-time points:

Full model	*state anxiety ~ time + time^2 + trait math anxiety + time : trait math anxiety + time^2 : trait math anxiety + (1 | subject)*	
Step 1	*state anxiety ~ time + time^2 + trait math anxiety + time^2 : trait math anxiety + (1 | subject)*	The model excluding the interaction of time and trait math anxiety was significantly different from the original model ($\chi^2 = 14.52$, $p < .001$). Therefore, we retained the interaction of time and trait math anxiety in the final model.
Step 2	*state anxiety ~ time + time^2 + trait math anxiety + time : trait math anxiety + (1 | subject)*	The model excluding the interaction of time² and trait math anxiety was not significantly different from the original model ($\chi^2 = 0.90$, $p = .344$). Therefore, we removed the interaction of time² and trait math anxiety in the final model.
Step 3	*state anxiety ~ time + trait math anxiety + time : trait math anxiety + (1 | subject)*	The model excluding the fixed effect of time² was not significantly different from the original model ($\chi^2 = 1.92$, $p = .382$). Therefore, we removed the fixed effect of time² in the final model.
Final Model	**Model S1**: *state anxiety ~ time + trait math anxiety + time : trait math anxiety + (1 | subject)*	Final model = Step 3
Random effects	subject (intercept, variance = 0.16, SD = 0.40) (residual, variance = 0.17, SD = 0.41)	

4. State (math) anxiety across six-time points

(1) LMM for state math anxiety across six-time points:

Full model	*state math anxiety ~ time + time^2 + trait math anxiety + time : trait math anxiety + time^2 : trait math anxiety+ (1 | subject) + (1 | paradigm)*	
Step 1	*state math anxiety ~ time + time^2 + trait math anxiety + time : trait math anxiety + time^2 : trait math anxiety+ (1 | subject)*	The model excluding the random intercept of paradigm was significantly different from the original model ($\chi^2 = 80.18$, $p < .001$). Therefore, we retained the random intercept of paradigm in the final model.

Step 2	*state math anxiety ~ time + time^2 + trait math anxiety + time^2 : trait math anxiety+ (1 | subject) + (1 | paradigm)*	The model excluding the interaction of time and trait math anxiety was significantly different from the original model ($\chi^2 = 15.45$, $p < .001$). Therefore, we retained the interaction of time and trait math anxiety in the final model.
Step 3	*state math anxiety ~ time + time^2 + trait math anxiety + time : trait math anxiety + (1 | subject) + (1 | paradigm)*	The model excluding the interaction of time^2 and trait math anxiety was not significantly different from the original model ($\chi^2 = 0.63$, $p = .428$). Therefore, we removed the interaction of time^2 and trait math anxiety in the final model.
Step 4	*state math anxiety ~ time + trait math anxiety + time : trait math anxiety + (1 | subject) + (1 | paradigm)*	Remove the main effect of time^2. ANOVA showed it was not significant ($\chi^2 = 0.81$, $p = .668$). Removed.<br>The model excluding the main effect of time^2 was not significantly different from the original model ($\chi^2 = 0.81$, $p = .668$). Therefore, we removed the main effect of time^2 in the final model.
Final model	**Model E**: *state math anxiety ~ time + trait math anxiety + time : trait math anxiety + (1 | subject) + (1 | paradigm)*	Final model = Step 4
Random effects	subject (intercept, variance = 0.33, SD = 0.57)<br>paradigm (intercept, variance = 0.06, SD = 0.24)<br>(residual, variance = 0.17, SD = 0.41)	

(2) LMM for state anxiety across six-time points:

Full model	*state anxiety ~ time + time^2 + trait math anxiety + time : trait math anxiety + time^2 : trait math anxiety + (1 | subject) + (1 | paradigm)*	
Step 1	*state anxiety ~ time + time^2 + trait math anxiety + time : trait math anxiety + time^2 : trait math anxiety + (1 | subject)*	The model excluding the random intercept of paradigm was significantly different from the original model ($\chi^2 = 108.58$, p < .001). Therefore, we retained the random intercept of paradigm in the final model.
Step 2	*state anxiety ~ time + time^2 + trait math anxiety + time^2 : trait math anxiety + (1 | subject) + (1 | paradigm)*	The model excluding the interaction of time and trait math anxiety was not significantly different from the original model ($\chi^2 = 1.90$, $p = .168$). Therefore, we removed the interaction of time and trait math anxiety in the final model.
Step 3	*state anxiety ~ time + time^2 + trait math anxiety +*	The model excluding the interaction of

	(1 | subject) + (1 | paradigm)	$time^2$ and trait math anxiety was significantly different from the original model ($\chi^2 = 6.33$, $p = .042$). Therefore, we retained the interaction of $time^2$ and trait math anxiety in the final model.
Step 4	*state anxiety ~ time^2 + trait math anxiety + time^2 : trait math anxiety + (1 | subject) + (1 | paradigm)*	Remove the main effect of time.<br>The model excluding the main effect of time was significantly different from the original model ($\chi^2 = 55.30$, $p < .001$). Therefore, we retained the main effect of time in the final model.
Final model	**Model S2:** *state anxiety ~ time + time^2 + trait math anxiety + time^2 : trait math anxiety + (1 | subject) + (1 | paradigm)*	Final model = Step 2
Random effects	subject (intercept, variance = 0.27, SD = 0.52)<br>paradigm (intercept, variance = 0.08, SD = 0.28)<br>(residual, variance = 0.16, SD = 0.40)	

5. Anxiety-complexity effect for response time but not accuracy

(1) LMM for response time:

Full model	*response time ~ trait math anxiety + difficulty + trait math anxiety : difficulty + (1 + paradigm | subject) + (1 | item)*	
Step 1	*response time ~ trait math anxiety + difficulty + trait math anxiety : difficulty + (1| subject) + (1 | item)*	The model excluding the random slope for paradigm was significantly different from the original model ($\chi^2 = 722.34$, $p < .001$). Therefore, we retained the random slope for paradigm in the final model.
Step 2	*response time ~ trait math anxiety + difficulty + trait math anxiety : difficulty + (1 + paradigm | subject)*	The model excluding the random intercept for item was significantly different from the original model ($\chi^2 = 1748.60$, $p < .001$). Therefore, we retained the random intercept for item in the final model.
Step 3	*response time ~ trait math anxiety + difficulty + trait math anxiety : difficulty + (1 + paradigm | subject) + (1 | item)*	The model excluding the interaction of trait math anxiety and difficulty was significantly different from the original model ($\chi^2 = 29.77$, $p < .001$). Therefore, we retained the interaction of trait math anxiety and difficulty in the final model.
Final Model	**Model S3**: *response time ~ trait math anxiety +*	Final model = Full model

	difficulty + trait math anxiety : difficulty + (1 + paradigm | subject) + (1 | item)	
Random effects	item (intercept, variance = 0.28, *SD* = 0.53)<br>subject (intercept, variance = 0.65, *SD* = 0.81)<br>paradigm (slope, variance = 0.33, *SD* = 0.57, *r* = .06)<br>(residual, variance = 2.10, *SD* = 1.45)	

(2) GLMM for accuracy:

Full model	*accuracy ~ trait math anxiety + difficulty + trait math anxiety : difficulty + (1 + paradigm | subject) + (1 | item)*	
Step 1	*accuracy ~ trait math anxiety + difficulty + trait math anxiety : difficulty + (1 | subject) + (1 | item)*	The model excluding the random slope for paradigm was significantly different from the original model (χ^2 = 78.40, $p < .001$). Therefore, we retained the random slope for paradigm in the final model.
Step 2	*accuracy ~ trait math anxiety + difficulty + trait math anxiety : difficulty + (1 + paradigm | subject)*	The model excluding the random intercept for item was significantly different from the original model (χ^2 = 97.85, $p < .001$). Therefore, we retained the random intercept for item in the final model.
Step 3	*accuracy ~ trait math anxiety + difficulty + (1 + paradigm | subject) + (1 | item)*	The model excluding the interaction of trait math anxiety and difficulty was not significantly different from the original model (χ^2 = 0.36, p = .550). Therefore, we removed the interaction of trait math anxiety and difficulty in the final model.
Step 4	*accuracy ~ difficulty + (1 + paradigm | subject) + (1 | item)*	The model excluding the main effect of trait math anxiety was not significantly different from the original model (χ^2 = 4.48, p = .107). Therefore, we removed the main effect of trait math anxiety in the final model.
Step 5	*accuracy ~ (1 + paradigm | subject) + (1 | item)*	The model excluding the main effect of difficulty was significantly different from the original model (χ^2 = 93.59, $p < .001$). Therefore, we retained the main effect of difficulty in the final model.
Final Model	**Model S4**: *accuracy ~ difficulty + (1 + paradigm | subject) + (1 | item)*	Final model = Step 4
Random	item (intercept, variance = 0.16, SD = 0.40)	

effects	subject (intercept, variance = 0.30, SD = 0.55)<br>paradigm (slope, variance = 0.22, SD = 0.46, r = -0.11)	

6. No speed-accuracy trade-off

Full model	*accuracy ~ paradigm + trait math anxiety + response time + paradigm : trait math anxiety + paradigm: response time + trait math anxiety : response time + paradigm : trait math anxiety : response time + (1 | subject)*	
Step 1	*accuracy ~ paradigm + trait math anxiety + response time + paradigm : trait math anxiety + paradigm: response time + trait math anxiety : response time + (1 | subject)*	The model excluding the three-way interaction was not significantly different from the original model (χ^2 = 0.30, p = .585). Therefore, we removed the three-way interaction in the final model.
Step 2	*accuracy ~ paradigm + trait math anxiety + response time + paradigm : trait math anxiety + trait math anxiety : response time + (1 | subject)*	The model excluding the interaction between paradigm and response time was not significantly different from the original model (χ^2 = 1.66, p = .435). Therefore, we removed the interaction between paradigm and response time in the final model.
Step 3	*accuracy ~ trait math anxiety + response time + trait math anxiety : response time + (1 | subject)*	The model excluding the interaction between paradigm and trait math anxiety was not significantly different from the original model (χ^2 = 1.75, p = .625). Therefore, we removed the interaction between paradigm and trait math anxiety in the final model.
Step 4	*accuracy ~ paradigm + trait math anxiety + response time + (1 | subject)*	The model excluding the interaction between trait math anxiety and response time was not significantly different from the original model (χ^2 = 4.38, p = .357). Therefore, we removed the interaction between trait math anxiety and response time in the final model.
Step 5	*accuracy ~ trait math anxiety + response time + (1 | subject)*	The model excluding the main effect of paradigm was significantly different from the original model (χ^2 = 25.22, p < .001). Therefore, we retained the main effect of paradigm in the final model.
Step 6	*accuracy ~ paradigm + response time + (1 | subject)*	The model excluding the main effect of trait math anxiety was not significantly

		different from the original model (χ^2 = 6.49, *p* = .262). Therefore, we removed the main effect of trait math anxiety in the final model.
Step 7	*accuracy ~ paradigm + (1 | subject)*	The model excluding the main effect of response time was not significantly different from the original model (χ^2 = 7.91, *p* = .245). Therefore, we removed the main effect of response time in the final model.
Final model	**Model S5**: *accuracy ~ paradigm + (1 | subject)*	Final model = Step 7
Random effects	subject (intercept, variance = 0.15, SD = 0.39)<br>(residual, variance = 0.08, SD = 1.45)	

Part 3: Exploratory analysis

Exploratory analysis 1: Correlation between state math anxiety and performance across paradigms

To examine whether performance might account for the observed paradigm effects on state math anxiety (SMA), we computed *Pearson* correlations between SMA and arithmetic performance, including response time (RT) and accuracy (ACC), across and within paradigms. As shown in Table S3, changes in SMA (ΔSMA) from decision to production paradigms were not significantly correlated with changes in RT, but showed a significant negative correlation with changes in ACC. This indicates that individuals who show higher increases of state math anxiety in production than decision paradigms are the ones who make more errors in production than decision paradigms. Within paradigms, SMA correlated negatively with accuracy but not with RT in production paradigms, whereas no significant correlations were found for decision paradigms. This indicates that higher state math anxiety is related to more errors in production paradigms. This pattern suggests that heightened SMA is more closely related to concerns about errors rather than slower processing.

Table S4. Exploratory correlation analyses between state math anxiety and performance across and within paradigms

Correlations	***r***	***95% CI***	***p***
ΔSMA and ΔRT	.18	[-.07, .41]	.153
ΔSMA and ΔACC	-.27	[-.49, -.03]	**.028**
SMA and RT (production)	.23	[-.02, .45]	.070
SMA and ACC (production)	-.26	[-.48, -.02]	**.034**
SMA and RT (decision)	.17	[-.08, .40]	.179
SMA and ACC (decision)	-.08	[-.32, .16]	.506

Exploratory analysis 2: Time-wise correlation between state math anxiety and performance

To further explore how the relationship between state math anxiety (SMA) and task performance evolved over time, we computed time-wise *Pearson* correlations between SMA and response time (RT) as well as logit-transformed accuracy (ACC) across the six measurement time points. For each time point, the correlation coefficient (r) and its 95% confidence interval were estimated using Fisher's z transformation (see Table S4 and Figure S3).

Overall, higher SMA tended to be associated with slower responses (positive SMA and RT correlation) and lower accuracy (negative SMA–ACC correlation). For RT, the strength of these associations decreased over time, suggesting that the coupling between anxiety and performance was strongest at the beginning of the experiment and attenuated as participants became more familiar with the task.

These exploratory findings indicate that state math anxiety exerts a transient impact on early performance before participants adapt to task demands.

Table S5. Correlation between state math anxiety and performance across six time points

Correlations	Time	*r*	*95% CI*	*p*
SMA and RT	1	.28	[.024, .499]	**.033**
	2	.34	[.105, .545]	**.006**
	3	.18	[-.076, .409]	.169
	4	.11	[-.149, .346]	.417
	5	.05	[-.212, .299]	.725
	6	-.07	[-.308, .185]	.610
SMA and ACC	1	-.08	[-.322, .166]	.516
	2	-.27	[-.481, -.027]	**.030**
	3	-.11	[-.348, .137]	.378
	4	-.27	[-.484, -.026]	**.031**
	5	-.20	[-.423, .050]	.117
	6	-.29	[-.502, -.054]	**.017**

Figure S3. Time-wise correlation between state math anxiety and performance

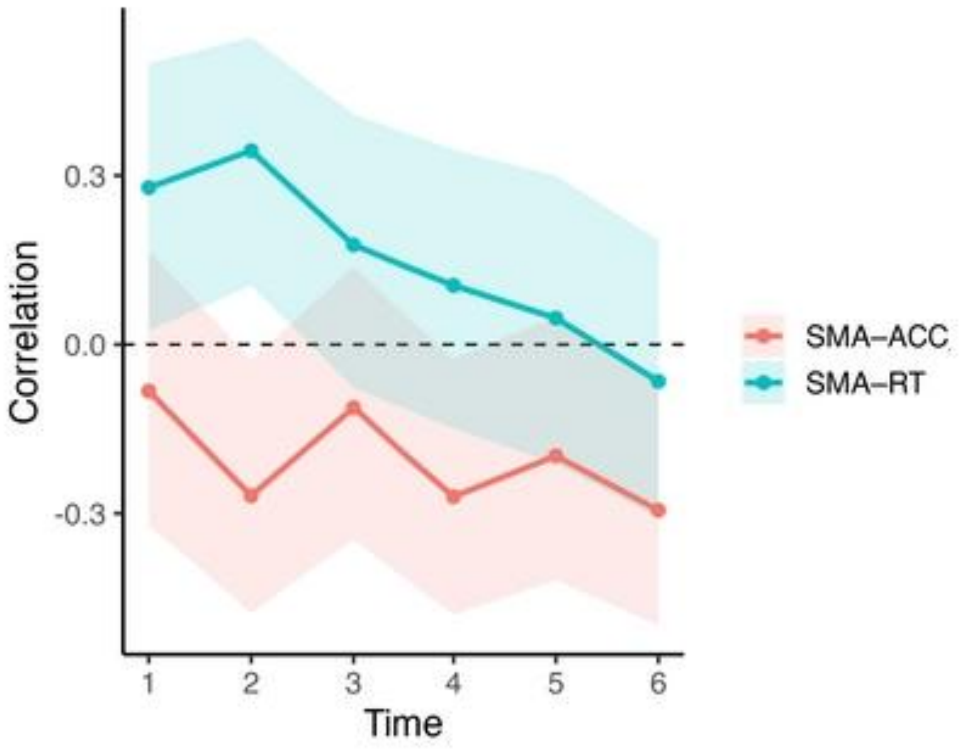

Notes. ACC represents accuracy, RT represents response time.
